# Rapid *Staphylococcus aureus* Detection From Clinical Mastitis Milk by Colloidal Gold Nanoparticle-Based Immunochromatographic Strips

**DOI:** 10.3389/fvets.2019.00504

**Published:** 2020-01-22

**Authors:** Yuya Nagasawa, Yoshio Kiku, Kazue Sugawara, Naohiro Yabusaki, Kazuyoshi Oono, Kento Fujii, Takahide Suzuki, Koji Maehana, Tomohito Hayashi

**Affiliations:** ^1^Dairy Hygiene Unit, Division of Pathology and Pathophysiology, Hokkaido Research Station, National Institute of Animal Health, National Agriculture and Food Research Organization, Sapporo, Japan; ^2^NOSAI Minami, Ebetsu, Japan; ^3^Hokubu Veterinary Clinic, Chiba P.F.A.M.A.A, Katori, Japan; ^4^Healthcare R&D Center, Asahi Kasei Corporation, Fuji, Japan

**Keywords:** bovine mastitis, immunochromatographic strip, milk, rapid diagnosis technology, *Staphylococcus aureus*

## Abstract

Rapid diagnostic technologies for bovine mastitis caused by *Staphylococcus aureus* (*S. aureus*) are urgently needed. In the current study, we generated an anti-ribosomal protein-L7/L12 antibody to detect *S. aureus* and an anti-ribosomal protein-L7/L12 antibody-coated immune-chromatographic strip (ICS) test. Moreover, we determined the ability of the ICS test to detect *S. aureus* from milk samples collected from cows with clinical mastitis. The developed ICS reacted to *S. aureus* in a bacteria load-dependent manner with a detection limit of ~10^4^ CFU/mL. In the evaluation of possible cross-reactivity of the ICS test, six strains of coagulase-negative Staphylococci showed slightly positive reactions, although at a lower level; however, other bacteria were completely negative. Next, we investigated the sensitivity and specificity of the ICS test compared with the bacteriological culture method using milk samples from clinical bovine mastitis. The results of the experiments demonstrated that the ICS test had high sensitivity [100%, 95% confidence interval (CI): 91.3–100%] and specificity (91.9%, CI: 90.5–91.9%) compared with culture tests. In addition, the kappa statistic demonstrated that ICS tests showed substantial agreement (k = 0.77, CI: 0.66–0.87) with culture tests. Positive correlations were observed for the statistical analysis between *S. aureus* (*nuc* gene) copy numbers and ICS test scores in mastitic milk infected by *S. aureus*. Therefore, we assume that this new detection method using ICS may be useful as a highly sensitive *S. aureus*-screening method for the diagnosis of bovine mastitis. Our findings support the ongoing effort to develop an ICS method for bovine *S. aureus*-induced mastitis, which can contribute to the rapid diagnosis of this disease.

## Introduction

*Staphylococcus aureus* (*S. aureus*) is one of the most significant bovine mastitis pathogens and is therefore related to major economic losses (1, 2). Because the treatment for bovine mastitis varies according the pathogen, successful clinical mastitis treatment relies on early detection and suitable diagnosis, including accurate identification of the pathogen involved (3). Indeed, in case of japan, guidebook of antibiotic treatment for bovine mastitis by Ministry of Agriculture, Forestry and Fisheries of Japan recommends that clinical cases in lactating cows should be treated with an appropriate intramammary antibiotic based on Roberson's recommendations (4). Especially for *S. aureus* which is a contagious mastitis pathogen, an efficient diagnostic assay would constitute a real progress for the improvement of herd management. Bacterial culture and isolate identification are the gold standard diagnostic methods for bovine mastitis diagnosis (5). However, bacterial identification using the culture process is complex and time consuming. Furthermore, even if colonies are obtained from culture, skilled technicians are still required for identification. Therefore, rapid diagnostic technologies for bovine mastitis caused by *S. aureus* are urgently needed.

Currently, mastitis pathogens diagnosis mainly relies on bacteriological methods and polymerase chain reaction (PCR) assays. The diagnostic accuracy of PCR-based methods has shown high sensitivity and specificity in detection of bacteria in milk, compared to conventional bacterial culture for microbes such as *S. aureus* and *Streptococcus uberis* (6). In addition, in clinical mastitis milk samples, statistical analysis with kappa test confirmed very good agreement among culture method, the 16S rRNA partial genome sequence analysis and the Matrix Assisted Laser Desorption/Ionization results for identifying the main mastitis pathogens (7). However, in general, genotypic methods require investment in equipment that is usually very expensive, which limits their use in routine diagnosis. Concerning ELISA and other immunological methods, they are currently not used with bovine mastitis milks because of their high detection limit and a lack of sensitivity and specificity.

Ribosomal protein (RP)-L7/L12 belongs to the 50S ribosome, which is richly expressed in many microbes. RP-L7/L12 contains specific sequences for individual bacterial species (8, 9). In addition, because RP-L7/L12 is essential for protein synthesis in microbes, RP-L7/L12 levels increase in proportion of the bacterial growth rate (10). Similar proteins are found in the large ribosomal subunits of archaebacteria, eukaryotes, and all eubacteria. Although archaebacterial and eukaryotic proteins are homologous, they show little homology to eubacterial proteins, as assessed by various physical and functional criteria (11). Thus, RP-L7/L12 is highly specific for each bacterium and can be useful as a target for rapid diagnosis.

Lateral flow tests, also known as immune-chromatographic strip (ICS) tests, are rapid tests that can reduce the time spent waiting for test results from hours to minutes utilizing classical immunochromatographic assays. These tests require no specialized equipment nor technical training for operators. Thus, ICS tests are suitable for on-site testing (12). Previous studies have reported the rapid diagnostic usefulness of RP-L7/L12 as a target for the diagnosis of *Streptococcus pneumoniae* and *Mycoplasma pneumoniae* infection by ICS tests (13, 14). These results have suggested that ICS tests targeting bacterial RP-L7/L12 could be useful for the rapid diagnosis of a variety of infectious diseases, if specific monoclonal antibodies (mAbs) become available for the detection of certain bacterial RP-L7/L12. Therefore, we assumed that an ICS test incorporating *S. aureus* anti-RP-L7/L12 protein may be effectively utilized as a novel method to identify *S. aureus* in milk from cows with bovine mastitis.

Accordingly, in this study, we generated an anti-RP-L7/L12 monoclonal antibody to detect *S. aureus* and developed anti-RP-L7/L12 antibody-coated ICS tests. Moreover, we determined the ability of the ICS test to detect *S. aureus* from milk samples collected from cows with clinical mastitis. Appropriate treatment of clinical mastitis on each farm is an important factor for improving the effectiveness of mastitis prevention programs to control infectious pathogens (15, 16). Recurrent *S. aureus* infections are generally difficult to cure during the lactation period and their medical management is different from clinical mastitis. Indeed, the best option for these chronic mastitis is to milk the affected cows last and wait for the antibiotic treatment at drying off and/or eliminate infected cows to prevent spread within the herd (17). Therefore, the present study focused on milk samples from the initial step of clinical mastitis, period associated with higher cure rate due to rapid diagnosis. Our findings provided a sensitive and rapid method for detection of bovine mastitis caused by *S. aureus*.

## Materials and Methods

### Preparation of Anti-RP-L7/L12 mAbs

**Figure 1:**
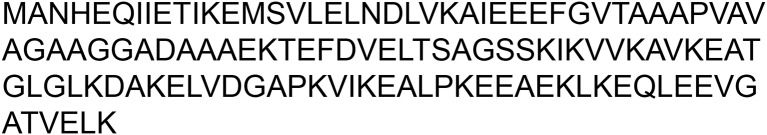
Amino acid sequence of full-length RP-L7/L12 (GeneBank accession no. OOC91413.1).

**Figure 2:**
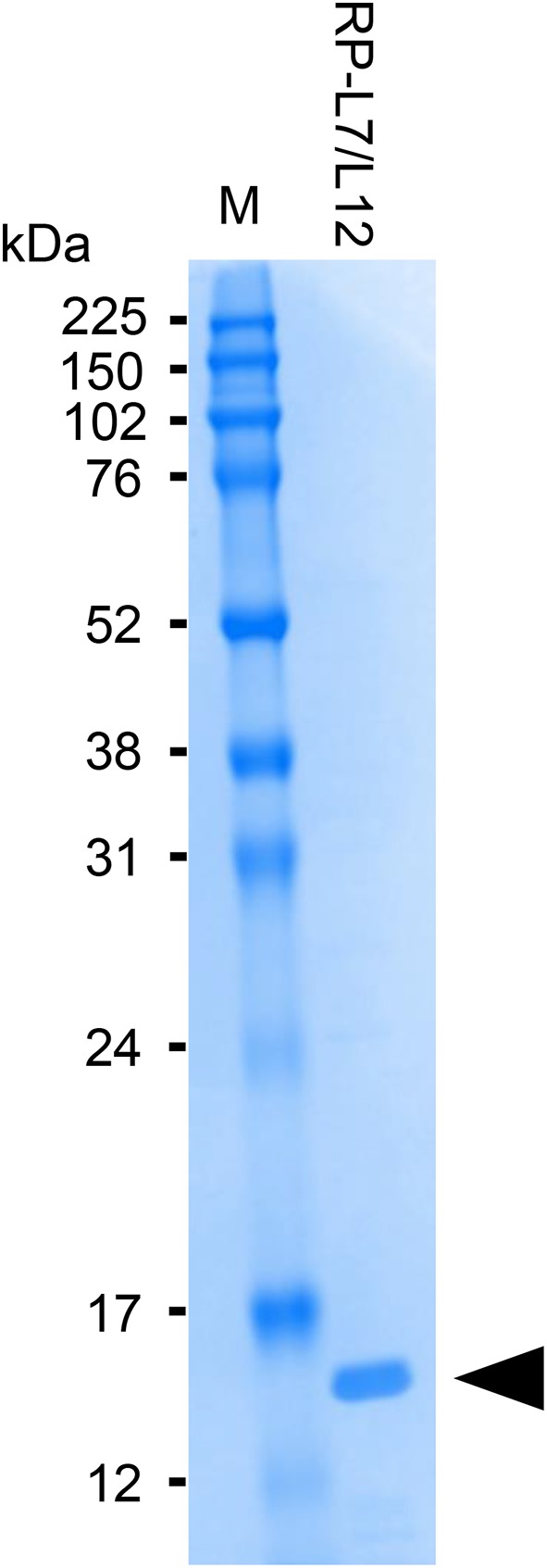
SDS-PAGE analysis of purified RP-L7/12. Coomassie brilliant blue-stained gel showing RP-L7/L12 after removal of the GST-tag (arrow head; molecular mass of RP-L7/L12 = ~13 kDa). M represents the molecular weight marker.

The anti-RP-L7/L12 mAbs used for manufacturing ICS were prepared by conventional methods described by Sano et al. (13). Briefly, RP-L7/L12 was amplified by PCR using genomic DNA from *S. aureus* (ATCC25923) as template and the following oligonucleotide primers: forward, 5′-CTAGGATCCATGGCTAATCATGAACAAATC-3′, and reverse 5′-CTAGAATTCTTATTTTAATTCTACAGTAGCTCCAAC-3′. The PCR product was cut with BamHI and EcoRI and then inserted into pGEX-6P-1 vector (GE Healthcare, Tokyo, Japan) digested with the same restriction enzymes. The recombinant plasmid was transformed into *Escherichia coli* BL21 cells carrying bacteriophage DE3 for protein overexpression. The recombinant protein was then purified on an affinity column, and cleaved to yield the final protein by enzyme removal of the glutathione S-transferase tag (PreScission Protease; GE Healthcare). Recombinant RP-L7/L12 is composed of 122 amino acids, as shown in Figure 1. The purity of the recombinant RP-L7/L12 protein was checked by Sodium dodecyl sulfate (SDS)-polyacrylamide gel electrophoresis (PAGE) with SDS-Tris-glycine running buffer system and with Coomassie brilliant blue staining, showing a 13 kDa protein (Figure 2).

The purified anti-RP-L7/L12 mAbs were obtained by conventional method (18) and were prepared by Asahi Kasei Corporation, Japan. Briefly, mice were intraperitoneal injected with 10 μg purified RP-L7/L12 with a 2-week interval. Six weeks later, 1.0 × 10^8^ cells of spleen cells were isolated and fused to 2 × 10^7^ cells of the myeloma cell line NS-1 using polyethylene glycol. The supernatants were screened by ELISA against RP-L7/L12 as described below. The wells that exhibited immunospecificity to the targets were subjected to cloning by limiting dilution to obtain monoclonal cell populations. RP-L7/L12 mAbs were purified from supernatant harvested from RP-L7/L12-secreting mouse hybridoma cells by Protein G column. Purified RP-L7/12 mAbs SA-1 and SA-2 were stored at −30°C.

**Figure 3:**
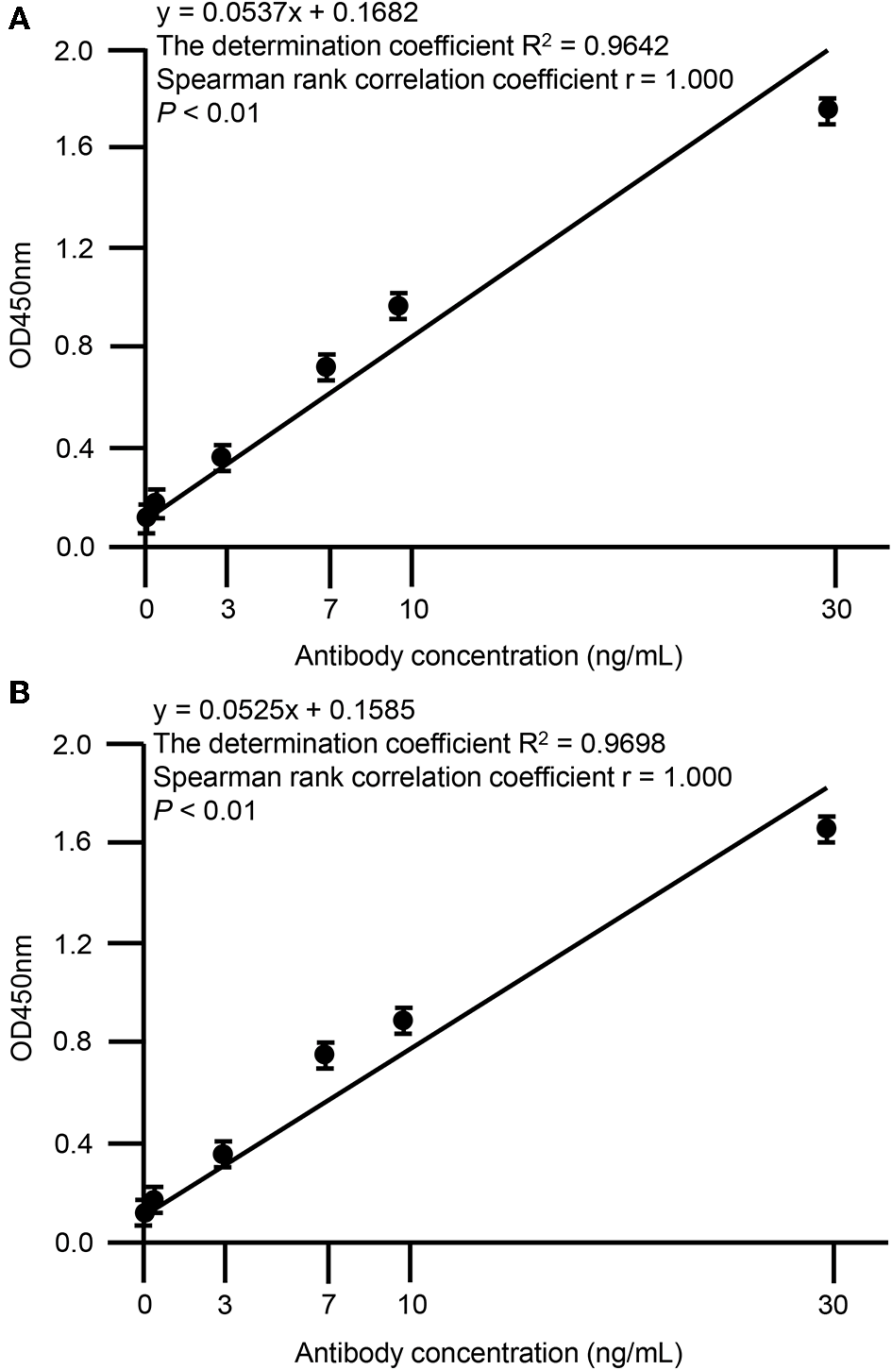
Correlation between OD values of anti-RP-L7/L12 mAbs and dose-dependency. Statistical correlations among the clones SA-1 **(A)** or SA-2 **(B)** and dose-dependency were evaluated by indirect ELISA. Each data point represents the mean value (±standard deviation) of the corresponding triplicates. Linear regression analysis of OD values and antibody concentrations is shown. A *P* < 0.01 was considered statistically significant.

Screening of hybridoma cells supernatants and binding ability of SA-1 and SA-2 mAbs were performed by ELISA. The 96 well ELISA microplates were coated overnight at 4°C with 50 μl per well of a 1 μg/mL RP-L7/L12 solution in phosphate-buffered saline (PBS)/0.05% (w/v) NaN3. After incubation, wells were washed with PBS-Tween-20 (PBST) and then incubated with 200 μL of 1% bovine serum albumin (BSA) for 2 h at room temperature (RT), and then hybridoma supernatants or serial dilutions of 0, 0.1, 1, 3, 7, 10, 30 μg/mL in PBS were added to the plates and incubated for 2 h at RT. After three PBST washes, wells were incubated with horseradish peroxidase-conjugated anti-mouse IgG antibody produced in goat (diluted 1:2000, Sigma-Aldrich, St. Louis, MO, USA) for 1 h at RT. The freshly prepared substrate using the 3′,3′,5,5′-tetramethylbenzidine microwell peroxidase substrate system (KPL, Gaithersburg, MD, USA) was added and optical density (OD) was measured at 450 nm by microplate reader (Spectra MAX 190, Molecular Devices, Sunnyvale, CA, USA). All samples were analyzed in triplicate and mean values were calculated. The OD values of SA-1 (Spearman rank correlation coefficient *r* = 1.000, *P* < 0.01) and SA-2 (Spearman rank correlation coefficient *r* = 1.000, *P* < 0.01) showed strong positive correlation with the dose dependent levels (Figure 3). The determination coefficient of SA-1 (*R*^2^ = 0.9642) and SA-2 (*R*^2^ = 0.9698) showed that the data obtained by ELISA represented a reliable basis (Figure 3).

### Development of the ICS

**Figure 4:**
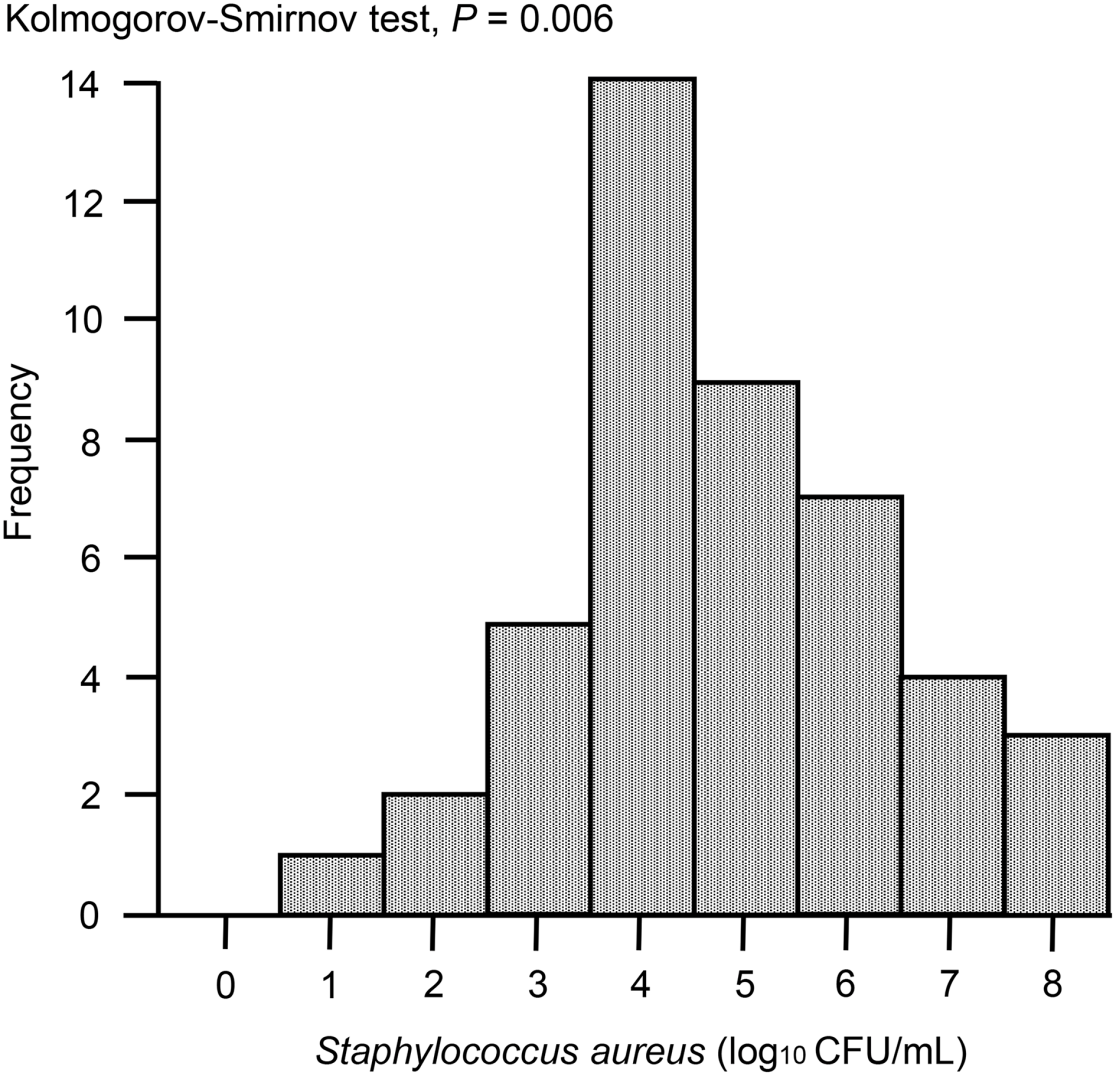
Histogram of *S. aureus* loads in milk samples collected from clinical mastitis. Forty seven milk samples from Holstein dairy cows with new infection of clinical mastitis were collected. The histogram shows the distribution of bacterial loads in milk. *P* < 0.05 were considered non-normal distribution across the analyzed group.

In the case of immunoassay tests, high sensitivity is desirable; however, there is also an increased possibility of false-positive results. Therefore, before development of the ICS tests, apart from milk samples for study of ICS test, 47 milk samples from Holstein dairy cows at the onset of clinical mastitis were collected from dairy farms located in Ishikari district, Hokkaido, Japan. According to the method described below, *S. aureus* bacterial loads in milk samples were measured using Petrifilm Staph Express Count plates (3M, Minneapolis, MN, USA). The distribution of *S. aureus* bacterial loads was analyzed visually using histograms, and descriptive statistics were produced accordingly. The distribution of *S. aureus* bacterial loads was skewed, as demonstrated by Kolmogorov-Smirnov tests, owing to the presence of large values (*P* = 0.006). When the *S. aureus* bacterial load was >10^3^ colony-forming units (CFU)/mL, ~90% of samples were distributed (Figure 4). Therefore, for the ICS tests used in this study, we adjusted the detection limit to a level of ~10^4^ CFU/mL, although it is no more than ~80% of clinical milk samples that were distributed with this threshold.

**Figure 5:**
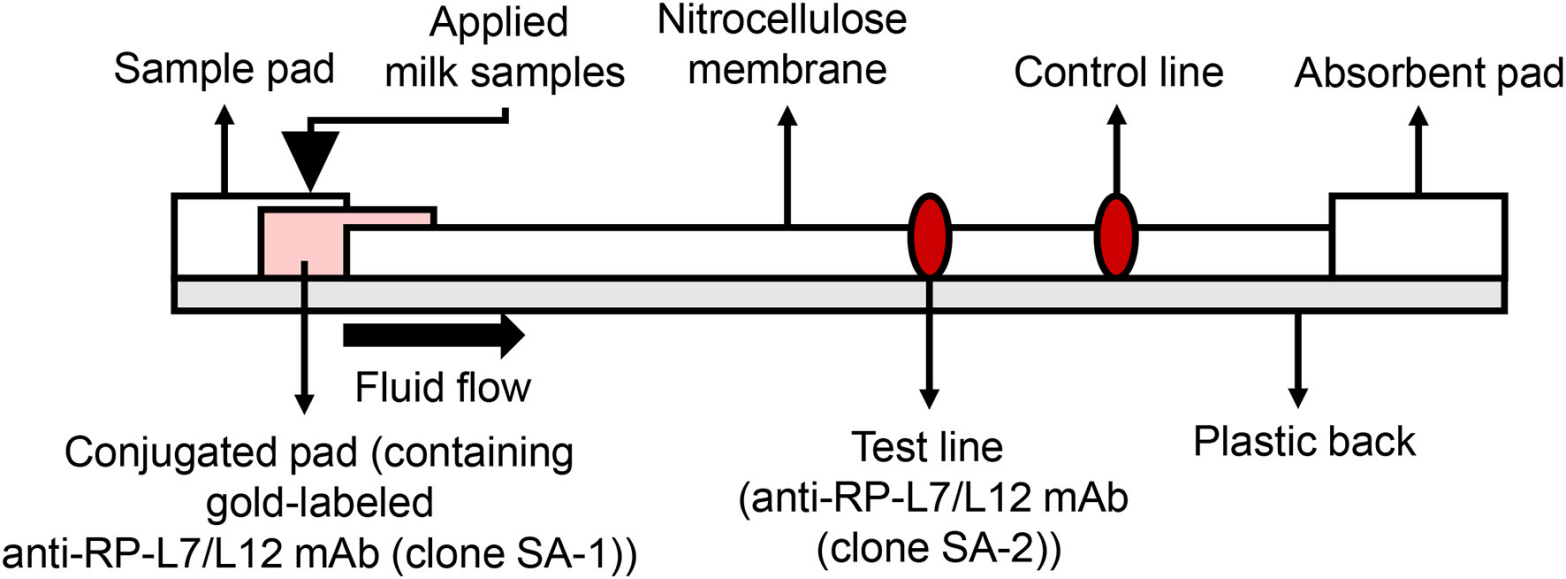
Description of the anti-RP-L7/L12 mAb-coated immunochromatographic strips.

Development of the ICS was carried out based on the patent US20150160212A1 (19). In the current study, we used colloidal gold-labeled antibodies, which are widely used for constructing ICS tests, to detect *S. aureus* RP-L7/L12. The 900 μL of colloidal gold solution (60 nm diameter, BBI solutions, Cardiff, UK) and 100 μL of SA-1 at final concentration of 2 mg/mL were mixed for 5 min at RT. Subsequently, BSA solution at final concentration of 1% (w/v) was added and mixed to block the excess reaction of the gold. After centrifugation for 5 min at 15,000 rpm and removal of the supernatant, the resulting pellet was suspended in 2 mL of 20 mM Tris-HCl buffer (pH 8.2)/0.25% (w/v) BSA, 2.5% (w/v) sucrose, 35 mM NaCl. The gold-labeled SA-1 solution was spotted onto the conjugated pad by vacuum drying at RT. At the test line, 1.5 mg/mL of SA-2 was spotted at 1 μL/cm at a position of 10 mm onto the 25 mm × 300 mm nitrocellulose membrane (0.22 μm pore size membrane, Sartorius, Göttingen, Germany); at the control line, rabbit anti-mouse IgG antibody was spotted, followed by drying the membrane at 50°C for 30 min, and then the nitrocellulose membrane was incubated overnight in 0.5% (w/v) sucrose buffer. Finally, all the components of the strip were laminated on a sheet with plastic back, including the sample pad, the conjugate pad, nitrocellulose membrane, and the absorbent pad (Figure 5). To break up peptidoglycan layers, 300 μL aliquots of milk samples were incubated with 500 μL extraction buffer containing 1% (v/v) Tween 20 (BioRad, Hercules, CA, USA), 0.1% (w/v) Zwittergent 3-12 (Calbiochem, San Diego, CA, USA) and 10 μg/ mL lysostaphin (Wako, Tokyo, Japan) for 30 min at RT, and the test strips were then dipped into the milk samples to allow the *S. aureus* antigen RP-L7/L12 and the gold-labeled clone SA-2 detection antibody to react. The resulting immunocomplexes mobilized by capillary action to the nitrocellulose membrane and were trapped by the capture mAb derived from the clone SA-2 on the strip. If the RP-L7/L12 is present in the capillary solution, the test line responds red. The test line did not turn red in the absence of RP-L7/L12. Rabbit anti-mouse IgG antibody immobilized on the control line captured excessive gold passing through the test line, causing red color to develop on the strip. This phenomenon indicated that the liquid sample successfully flowed the nitrocellulose membrane as positive control.

To confirm dose-dependent reaction to *S. aureus* by anti-RP-L7/L12 mAbs-coated ICS tests, we investigated the reactivity of the ICS tests at different *S. aureus* concentrations. *S. aureus* strain ATCC 25923 was incubated on tryptic soy medium solution (Becton, Dickinson and Company, Franklin Lakes, NJ, USA) at 37°C for 24 h, and the solution was then prepared with concentrations ranging from 0 to 5 × 10^5^ CFU/mL mixed with conventional milk (serial dilution: 0, 5 × 10^3^, 1 × 10^4^, 5 × 10^4^, 1 × 10^5^, 5 × 10^5^ CFU/mL). These samples were also incubated in extraction buffer containing detergent at RT for 30 min, and test strips were then dipped into the samples at RT for 30 min. The results of ICS were recorded with a digital camera. The pictures were imported into a computer, and image analysis software CS Analyzer 4 (ATTO, Tokyo, Japan) was used to analyze the images to obtain the corresponding signal intensity of the test line. Each concentration was tested at 3 times.

Furthermore, we evaluated the crossreactivity with other bacteria that may cause bovine mastitis in the newly developed *S. aureus* ICS test. Test assays using each *S. aureus* strain or other bacteria were carried out by mixed with conventional milk. These samples were also incubated in extraction buffer containing detergent at RT for 30 min, and test strips were then dipped into the samples at RT for 30 min. The presence or absence and scoring of the red test line were read by the naked eye.

### Milk Sampling Procedure for Mastitis Detection on Dairy Farms

Milk samples were collected in 2017 and 2018 from Holstein dairy cows belonging to dairy farms located in Ishikari district, Hokkaido, Japan. At each milking, the cows were examined for mastitis signs by farm workers and/or veterinarians. Each time a clinical mastitis was noticed, a quarter milk sample was aseptically collected before the administration of antibiotic treatment. The effect of freezing and thawing milk samples on the performance of ICS test was evaluated and freezing had no influence on ICS test scores (data not shown). Therefore, milk samples were frozen, and sent to the Hokkaido Research Station, National Institute of Animal Health, and then stored at 4°C until culture method, PCR and ICS test analysis.

### Identification of *S. aureus* in Milk by the Bacteriological Culture Method and Measurement of *S. aureus* Counts

*S. aureus* was identified in milk samples by the culture method as follows. First, 50 μL of each sample was plated on sheep blood agar plate (Nissui Pharmaceutical, Tokyo, Japan). Then, after 24 h of incubation at 37°C, each petri dish was inspected for bacterial growth and *S. aureus* was identified according colony characteristics. We considered milk samples to be contaminated if three or more different bacterial species were clearly observed on these bacterial cultures, and these samples were excluded from this study, as described by Pinzón-Sánchez and Ruegg (20). To confirm that the bacteria on the plates were *S. aureus*, colonies on blood agar plates were applied to direct PCR amplification kit (RR180A, Takara, Kusatsu, Japan), following the manufacturer's instructions. All PCR products were sequenced at the authorized inspection agency. Obtained sequences were blasted with the GenBank database (http://www.ncbi.nlm.nih.gov/Blast; 16S ribosomal RNA gene sequences [Bacteria and Archaea]) for species or genus assignment. The highest homology sequence with the GenBank database was selected as the identified species or genus, and samples were then identified as *S. aureus* or other species.

To measure the *S. aureus* loads in the identified milk samples, we spread 1 mL of each milk sample on a Petrifilm Staph Express Count plate, which is known to be suitable for identifying mastitis pathogens (21–23), incubated the plates at 37°C for 24 h, and then counted the colonies. The CFU/mL was calculated using the number of bacteria. The milk samples were divided into groups according to their *S. aureus* load, as follows: CFU ≤ 10^4^, 10^4^ < CFU ≤ 10^5^, 10^5^ < CFU ≤ 10^6^, and 10^6^ < CFU.

### Quantification of *S. aureus nuc* Gene by Real-Time Quantitative PCR (qPCR)

Milk samples were incubated in 10 μg/mL lysostaphin at 37°C for 30 min, and genomic DNA extracted from milk was then purified from 100 μL of samples using a Qiagen DNA Mini Kit (Qiagen, Valencia, CA, USA), according to the manufacturer's instructions. Based on the literature (24), we used the *nuc* gene as a target for qPCR. To quantify DNA specific for *S. aureus*, two oligonucleotide primers and a probe for the *nuc* target gene were designed, amplifying a 166-bp fragment; the oligonucleotide primer and probe sequences were as follows: for *nuc*, forward primer 5′-CCTGAAGCAAGTGCATTTACGA-3′, reverse primer 5′-CTTTAGCCAAGCCTTGACGAACT-3′, and probe 5′-CATCAGCATAAATATACGCTAAGCCACGTCCA-3′. The probe was labeled at the 5′ end with 6-carboxyfluorescein and at the 3′ end with 6-carboxytetramethylrhodamin (Microsynth). The reaction mixture contained 5 μL purified template DNA, 2 μL probe, 4 μL LightCycler TaqMan Master mix, 2 μL primers, and 5 μL Rnase/DNase-free water, resulting in a reaction volume of 20 μL. All reactions were performed in triplicate. The qPCR conditions were as follows: 95°C for 5 min, followed by 45 cycles of 95°C for 10 s, 55°C for 30 s, and 72°C for 1 s. Cycling was performed using a LightCycler 480 instrument (Roche Diagnostics, Basel, Switzerland). The copy number of the *nuc* gene was determined by assuming that, based on the size of the 2.7 to 2.8 Mbp genome of *S. aureus*, 1 ng of DNA equals 6 × 10^5^ times the entire genome and that the *nuc* gene is a single-copy gene (25, 26). Sensitivity, specificity, and kappa statistics of PCR was compared with those of the culture method.

### Application of the ICS Test for Field Clinical Samples

Milk samples were incubated in extraction buffer containing detergent at RT for 30 min as previously described, and test strips were then dipped into the samples. The presence or absence and scoring of the red test line were read by the naked eye after 30 min. Sensitivity, specificity, and kappa statistics of ICS tests were compared with those of the culture method.

### Statistical Analysis

Statistical analysis was conducted using SPSS software (IBM SPSS Statistics version 25, Tokyo, Japan). Statistical analyses of signal intensity of the test line were performed using one-way ANOVA followed by Scheffe's test to evaluate statistical differences among the bacterial load groups. A two-by-two table reflecting the results of the ICS test and culture tests, PCR and culture tests were generated. Prior to calculating overall sensitivity and specificity, a chi-square test for heterogeneity was performed to determine if it was legitimate to data in this study (27). Each sensitivity estimate was accompanied by exact 95% binomial confidence limits. The agreement between the results of the two tests was evaluated using kappa statistics with 95% confidence intervals (95% CIs). The interpretation of the kappa results was based on the proposal of Landis and Koch (28), as follows: poor (<0.00), slight (0.00–0.20), fair (0.21–0.40), moderate (0.41–0.60), substantial (0.61–0.80), and almost perfect (0.81–1.00). Spearman rank correlation coefficients and linear regression analyses were used to assess the relationships between *nuc* copy numbers and *S. aureus* loads, *nuc* copy numbers and ICS test scores. For ICS test scores, non-parametric Kruskal-Wallis tests were used to analyze differences among the *S. aureus* bacterial load groups. Differences with *P* < 0.05 were considered significant.

## Results

### Detection Limit and Crossreactivity of the ICS Test

**Figure 6:**
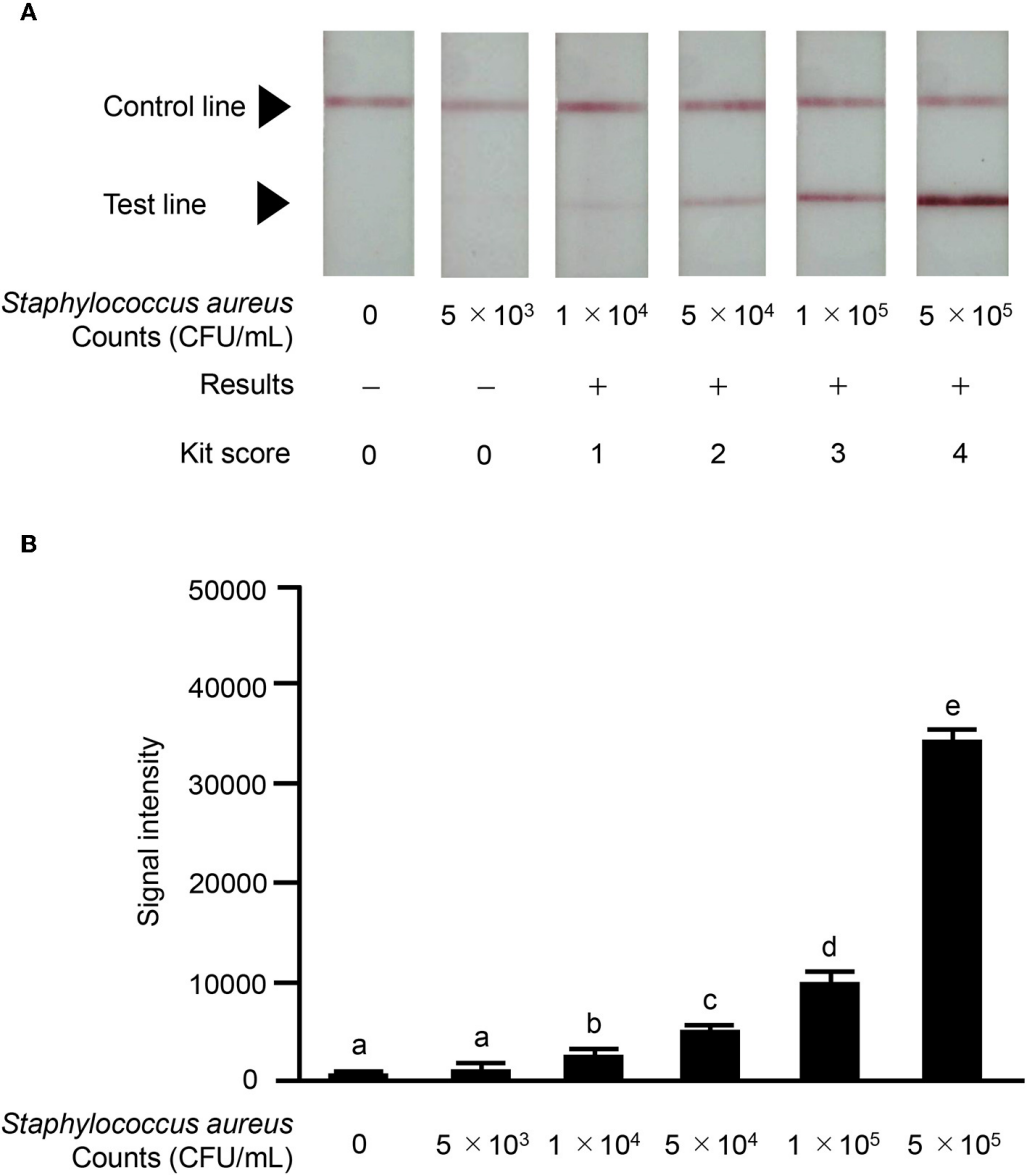
Results of ICS tests for *S. aureus* bacterial counts. Milk samples with *S. aureus* strain ATCC 25923: 0 CFU/mL; 5 × 10^3^ CFU/mL; 1 × 10^4^ CFU/mL; 5 × 10^4^ CFU/mL; 1 × 10^5^ CFU/mL; 5 × 10^5^ CFU/mL. **(A)** Negative result (–): no test line was observed. Positive result (+): the test line was observed. *S. aureus* counts corresponding to the scoring system for ICS tests were as follows: test line with no visible test line (score 0), with very weak reaction (score 1), darker than the control line (score 2), matching the control line (score 3), or lighter than the control line (score 4) were 1 × 10^4^, 5 × 10^4^, 1 × 10^5^, and 5 × 10^5^, respectively. **(B)** The signal intensities of the test line were analyzed by image analysis software CS Analyzer 4. Each bar graph represents the mean value (± standard deviation) of the corresponding triplicates. Letters (a, b, c, d, e) indicate a difference (*P* < 0.05) among groups, respectively.

We investigated the reactivity of the ICS test at different *S. aureus* concentrations. Reactions were observed with *S. aureus* concentrations of ≥1.0 × 10^4^ CFU/mL in a concentration-dependent manner, and strong reactions were observed with *S. aureus* concentrations of 5 × 10^5^ CFU/mL (Figure 6A). No significant differences in signal intensity of the test line could be detected between concentration of 0 CFU/mL and 5.0 × 10^3^ CFU/mL, but there was a significant difference among concentrations of 1 × 10^4^, 5 × 10^4^, 1 × 10^5^, and 5 × 10^5^ CFU/mL (Figure 6B). Based on this result, we developed a scoring system for ICS tests, with scores ranging from 0 to 4. The *S. aureus* loads corresponding to the ICS test scoring system were as follows: test line with no visible test line (score 0), with very weak reaction (score 1), darker than the control line (score 2), matching the control line (score 3), or lighter than the control line (score 4) indicated concentrations of 1 × 10^4^, 5 × 10^4^, 1 × 10^5^, and 5 × 10^5^ CFU/mL, respectively (Figure 6). Based on these ICS scores, in subsequent ICS tests, when the score was 1 or more, the result was judged as positive; scores of 0 were judged as negative. To explore the crossreactivity of the ICS test, one *S. aureus* strain, seven coagulase-negative *Staphylococci*, and 10 species of other bacterial pathogens associated with bovine mastitis were examined by ICS tests (Table 1). *Staphylococcus chromogenes, Staphylococcus sciuri, Staphylococcus hominis, Staphylococcus xylosus, Staphylococcus intermedius*, and *Staphylococcus lentus* showed positive results at a detection limit higher than that of *S. aureus* (a *Staphylococcus chromogenes* concentration of 1 × 10^7^ CFU/mL was a score of 2, a *Staphylococcus sciuri* concentration of 1 × 10^7^ CFU/mL was a score of 4, a *Staphylococcus hominis* concentration of 1 × 10^7^ CFU/mL was a score of 2, a *Staphylococcus xylosus* concentration of 1 × 10^7^ CFU/mL was a score of 3, a *Staphylococcus intermedius* concentration of 1 × 10^7^ CFU/mL was a score of 4 and *Staphylococcus lentus* concentration of 1 × 10^7^ CFU/mL was a score of 4); however, *Staphylococcus haemolyticus* was negative in the test. Additionally, the other 10 bacterial species (non *Staphylococcal* species) resulted in ICS score 0.

**Table 1:** ICS score obtained with different bacterial species.

**Bacteria species**	**Strain**	**Bacteria count (CFU)**	
		**1 × 10^**7**^**	**1 × 10^**6**^**
*Staphylococcus aureus*	ATCC25923	4	4
*Staphylococcus chromogenes*	Clinical isolate	2	0
*Staphylococcus haemolyticus*	Clinical isolate	0	0
*Staphylococcus sciuri*	Clinical isolate	4	1
*Staphylococcus hominis*	Clinical isolate	2	1
*Staphylococcus xylosus*	Clinical isolate	3	2
*Staphylococcus intermedius*	Clinical isolate	4	1
*Staphylococcus lentus*	Clinical isolate	4	1
*Corynebacterium lactis*	Clinical isolate	0	N/A
*Enterococcus faecalis*	ATCC19433	0	N/A
*Escherichia coli*	ATCC25922	0	N/A
*Klebsiella pneumoniae*	ATCC13883	0	N/A
*Mycoplasma bovis*	ATCC27368	0	N/A
*Pseudomoas aeruginosa*	ATCC27853	0	N/A
*Streptococcus agalactiae*	ATCC12386	0	N/A
*Streptococcus dysgalactia*	ATCC43078	0	N/A
*Streptococcus intermedius*	ATCC27335	0	N/A
*Streptococcus uberis*	ATCC19436	0	N/A

*N/A, not available*.

### Performance of the ICS Test With Clinical Milk Samples

In total, 258 milk samples from clinical mastitis were initially identified as eligible for the samples used in this study. After performing bacterial cultures, 12 samples were excluded due to contamination. The results of the bacteriological analysis performed with the milk samples are summarized in Table 2. Of the remaining 246 samples, 35 samples were identified as infected by *S. aureus* by the culture method. Thirty-five yielded colonies of *S. aureus* on Petrifilm Staph Express Count plates, and these samples were used for the subsequent analysis.

**Table 2:** Bacterial species identified in the clinical milk samples.

**Bacteria species or species group**	**Number of isolate**
Mixed infection-caused by two pathogens	25
*Escherichia coli*	64
*Streptococcus* sp.	43
*Staphylococcus aureus*	35
*Staphylococcus* sp.	25
*Klebsiella pneumoniae*	11
*Enterococcus* sp.	11
*Bacillus* sp.	5
*Ochrobactrum* sp.	5
*Pseudomonas* sp.	4
*Pantoea* sp.	3
*Escherichia* sp.	3
*Proteus* sp.	3
*Serratia* sp.	2
*Enterobactor* sp.	1
*Corynebacterium* sp.	1
*Obesumbacterium proteus*	1
*Brevibacterium linens*	1
*Raoultella terrigena*	1
*Lactococcus lactis*	1
*Aeromonas* sp.	1
Total	246

Thirty-five mastitic milk samples caused by *S. aureus* were culture positive, with all 35 also identified as positive in the RP-L7/L12 ICS test and qPCR for the gene *nuc* (sensitivity, 100.0%, CI: 91.3–100%, respectively). In contrast, 211 mastitic milk samples were culture negative for *S. aureus* growth, 194 of which were RP-L7/L12 ICS and PCR negative (specificity, 91.9%, CI: 90.5–91.9%, respectively). The kappa statistic demonstrated that the ICS test and qPCR for the gene *nuc* showed substantial agreement (k = 0.77, 95% CI: 0.66–0.87%, respectively) with the culture test (Table 3).

**Table 3:** Sensitivity, specificity, and kappa statistic of immunochromatography test strips and PCR.

		**Culture**		**Total**
		**Positive**	**Negative**	
**RP-L7/L12 ICS test**	**Positive**	35	17	52
	**Negative**	0	194	194
**Total**		35	211	246
Sensitivity (%): 100 (91.3–100)				
Specificity (%): 91.9 (90.5–91.9)				
Kappa statistic (95% Cl): 0.77 (0.66–0.87)				
**PCR**	**Positive**	35	17	52
	**Negative**	0	194	194
**Total**		35	211	246
Sensitivity (%): 100 (91.3–100)				
Specificity (%): 91.9 (90.5–91.9)				
Kappa statistic (95% Cl): 0.77 (0.66–0.87)				

### Quantification of the Bacterial Load in Clinical Samples by ICS Tests

**Figure 7:**
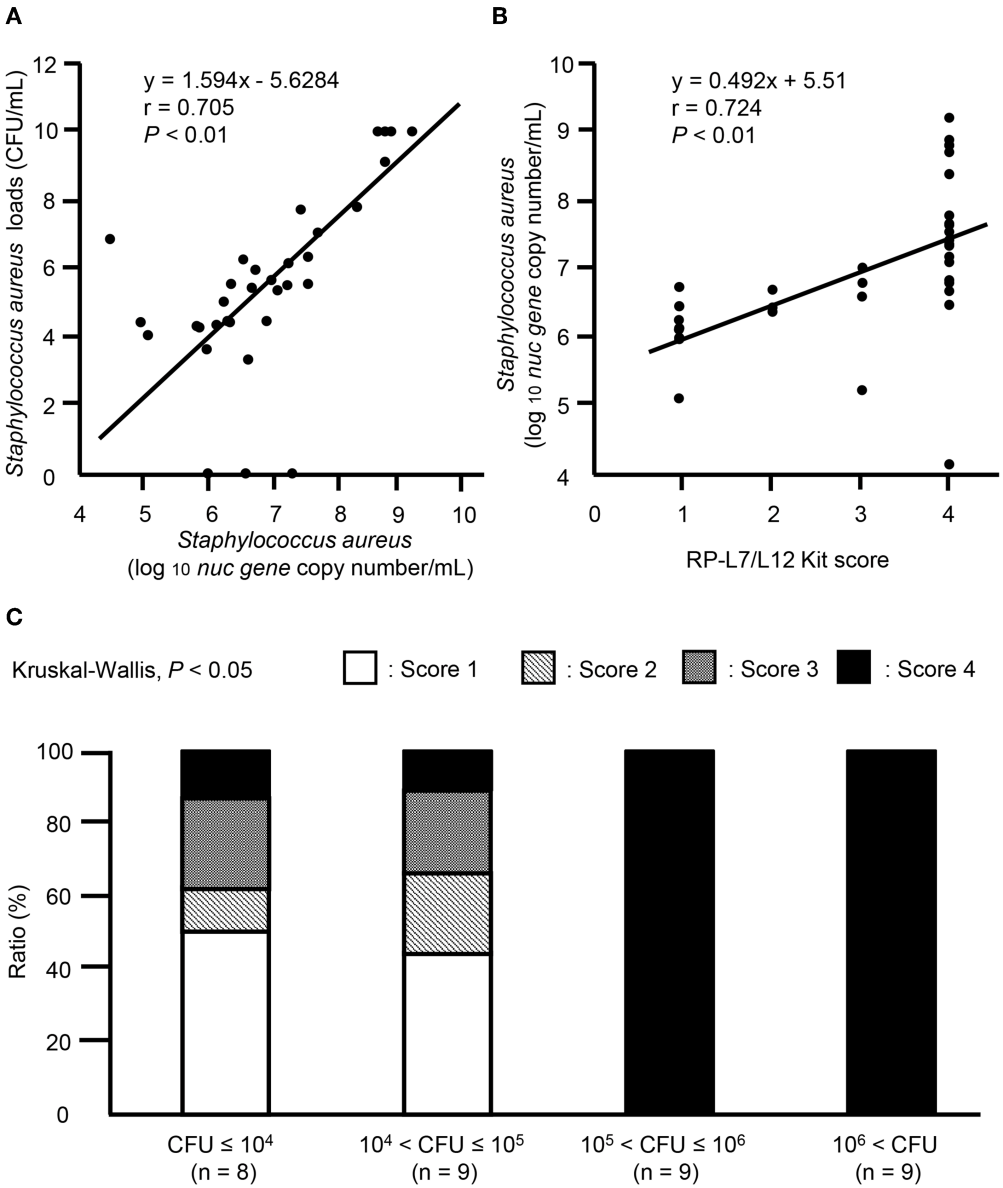
Evaluation of quantitative ICS test scores in clinical field samples. Correlation between **(A)** *nuc* gene copy numbers (log 10 *nuc* gene copy number/mL) and *S. aureus* loads (CFU/mL), **(B)** *nuc* gene copy numbers (log 10 *nuc* gene copy number/mL) and ICS test scores in milk samples. Evaluation of statistical correlations among *S. aureus* loads, *nuc* gene copy numbers, and ICS test scores in milk samples containing *S. aureus* (*n* = 35). Linear regression analysis of *nuc* gene copy numbers and *S. aureus* loads, *nuc* gene copy numbers and ICS test scores in milk samples containing *S. aureus* are shown. *P* < 0.05 were considered statistically significant. **(C)** Distribution of cows among ICS test scores and coliform bacteria load groups. Based on *S. aureus* bacteria loads, we divided 35 milk samples into groups, as follows: CFU ≤ 10^4^ (*n* = 8), 10^4^ < CFU ≤ 10^5^ (*n* = 9), 10^5^ < CFU ≤ 10^6^ (*n* = 9), and 10^6^ < CFU (*n* = 9). The stacked bar graph shows the distribution of cows in each *S. aureus* load group between the four different clinical severity grades: score 1 (white), score 2 (gray), score 3 (diagonal stripes), and score 4 (black). Values are given as number of cows (%). CFU, colony forming units.

In 35 mastitic milk samples positive for *S. aureus* by the culture method, ICS test scores ranged from 1 to 4; no scores of 0 were noted. To evaluate the quantitative scoring system for ICS tests, *nuc* gene copy numbers and ICS test scores were analyzed by Spearman rank correlation coefficients. Statistically positive correlations were observed between *nuc* gene copy numbers and *S. aureus* loads, between *nuc* gene copy numbers and ICS test scores in mastitic milk containing *S. aureus* (Figure 7A, *r* = 0.705, *P* < 0.01, Figure 7B, *r* = 0.724, *P* < 0.01). Then, we investigated the relationship between ICS test scores and *S. aureus* bacteria load. The ICS test scores in mastitis caused by *S. aureus* differed significantly among the *S. aureus* bacterial load groups (*P* < 0.05). When the *S. aureus* bacteria load higher than 10^5^ (CFU > 10^5^), 100% of milk samples had ICS test scores of 4 (Figure 7C).

## Discussion

In the current study, we established an ICS test for the rapid and accurate determination of bovine mastitis caused by *S. aureus* based on anti-RP-L7/L12 mAbs and anti-RP-L7/L12 mAb-coated ICSs. The developed anti-RP-L7/L12 mAb-coated ICS tests reacted to *S. aureus* in a bacteria load-dependent manner. Therefore, to examine efficacy of the anti-RP-L7/L12 mAb-coated ICS test for diagnosis of *S. aureus* infection in bovine mastitis, we investigated the sensitivity and specificity of the ICS test compared to bacteria culture method using clinical mastitis milk. The results of the experiments demonstrated that ICS tests had high sensitivity (100%, CI: 91.3–100%) and specificity (91.9%, CI: 90.5–91.9%) compared with culture tests. In addition, the kappa statistic analysis demonstrated that ICS tests showed substantial agreement (k = 0.77, 95% CI: 0.66–0.87) with the culture test. This strong agreement and the overall performance of the ICS test thus suggested that the ICS test was compatible with currently established bacteria culture methods. In particular, results from ICS tests could be obtained within 1 h after the arrival of the samples at the laboratory, compared with 1 day required for culture. In addition, because the freezing and thawing milk samples had no effect on results of ICS test scores, this ICS test could be carried out directly on the dairy farm by the farmer or a practitioner. Furthermore, ICS tests were easy to evaluate during routine diagnosis and strip tests are known to be easy to use, disposable test that do not introduce any contamination from previously tested samples (12, 29). Therefore, we believe that this new detection method using ICS tests may be useful as a highly sensitive *S. aureus*-screening method for the diagnosis of bovine mastitis. However, this ICS may need to be developed more conforming to the dairy farm situation as the test requires 30 min incubation in lysis buffer and measurement tools to dispense the correct volumes of milk and buffer.

Next, we evaluated the quantitative scoring system for ICS tests by investigating the relationship between *nuc* gene copy number, *S. aureus* bacterial load, and ICS test score. Positive correlations were observed for statistical analysis between *nuc* gene copy numbers and ICS test scores in mastitic milk containing *S. aureus*. qPCR can be used for quantitative analysis of bacterial loads in milk samples (24, 30). Indeed, for samples with *S. aureus* bacterial load of 10^5^ < CFU, 100% of milk samples had an ICS test score of 4, similar to dose-dependent *in vitro* tests. These results suggested that ICS test scores could be associated with bacterial load. However, this ICS will have value as a cut-off assay (above 1 × 10^4^ CFU) as with many lateral flow assays because there are small numbers of result with scores of 2 and 3 in this study. Additionally, bovine mastitis caused by *S. aureus* primarily causes subclinical intramammary infections that often progress to clinical infections. Subclinical mastitis is considered as the most economically important type of mastitis because of its higher prevalence and long-term devastating effects as compared to clinical mastitis (31). During *S. aureus* chronical mastitis, bacterial counts in milk could vary form 1 day to another with <1 × 10^4^ or 1 × 10^3^ CFU/mL. The ICS test may be integrated in a global mastitis control program and performed on a regular basis on quarter milk samples from cows displaying high somatic cell counts. Moreover, ICS test that adapts to subclinical mastitis might need to be more sensitive; however, the present study only focused on clinical mastitis. In future studies, to determine the applicability of the ICS test, it will be necessary to investigate the relationships between ICS test scores and clinical state, including subclinical or clinical, in more milk samples of bovine mastitis caused by *S. aureus*.

In this study, we demonstrated that anti-RP-L7/L12 mAb-coated ICS tests showed high sensitivity and specificity for detection of *S. aureus* in clinical mastitic milk compared with the bacteria culture method. However, the results of positive samples obtained from ICS tests and the bacterial culture method were mostly similar, although ICS tests tended to contain more false-positives. This phenomenon may be related to differences in the diagnostic principles of the tests. In the culture method, only alive bacteria will be detected. However, in the ICS test, which is based on an immunoassay, the anti-RP-L7/L12 antibody induces a positive response to killed bacteria. Indeed, these results could easily be inconsistent with the diagnosis results from the bacterial culture method because PCR has a potential for higher sensitivity and specificity due to its ability to detect both live and dead microorganisms (29, 32, 33). Actually, the present study also demonstrated that qPCR of *nuc* tended to contain false-positives at same level as ICS test. On the other hand, it should be noted that sensitivity and specificity of PCR is depends on target DNA. For example, Boss et al. demonstrated that high specificity and sensitivity were achieved by qPCR assay with 2 enterotoxins genes and a polymorphism within the leucotoxin E gene for *S. aureus* genotype B (34). Additionally, due to overgrowth of other pathogens in case of contamination of milk at sampling or mixed infection, the bacterial culture method may mask the actual pathogens causing mastitis. Another reason for inconsistent ICS tests may be the crossreactivity with coagulase-negative *Staphylococci*. Indeed, to confirm the specificity of the ICS test, we investigated the crossreactivity with 18 species of bacteria associated with mastitis. The results showed that *Staphylococcus chromogenes, Staphylococcus sciuri, Staphylococcus hominis, Staphylococcus xylosus, Staphylococcus intermedius*, and *Staphylococcus lentus* slightly reacted with the test strips, albeit at a lower level than *S. aureus*. Because of similarities in genetic backgrounds, some previous studies have shown crossreactivity with *S. aureus* (particularly methicillin-resistant *S. aureus*) and coagulase-negative *Staphylococci* in immunoassays. For example, incorporation of antibodies against capsule types 5 and 8 or against serotype 18 in several commercial agglutination tests can lead to false-positive reactions with coagulase-negative *Staphylococci* (35–37). Some *Staphylococcus* species other than *S. aureus* may expressed coagulase, pseudocoagulase, or surface proteins known to produce false-positive results with several latex kits. These cross reactivities may be due to incompletely purified antigen used for the preparation of monoclonal antibodies (38). Coagulase-negative *Staphylococci* can be present in some clinical samples, alone or with a major mastitis pathogen such as *S. aureus*. These false positive results by coagulase-negative *Staphylococci* should encourage the improvement of the ICS diagnostic assay to decrease the detection limit of *S. aureus* and increase the specificity of the test. Therefore, definite diagnosis of bovine mastitis by *S. aureus* should be carried out using the developed ICS tests in combination with culture tests and PCR tests. It is important to develop ICS tests that can accurately detect false-positive reactions in the dairy field.

In summary, we found that anti-RP-L7/L12 mAbs in milk were well-correlated with *S. aureus* bacterial loads. In addition, although six coagulase-negative *Staphylococci* strains were shown to have slightly positive reactions with the *S. aureus*, ICS tests measuring *S. aureus*-derived RP-L7/L12, which we developed as a new detection method, enabled efficient and rapid assessment of *S. aureus* in bovine clinical mastitis. The developed ICS test was rapid, simple, and convenient and could be used by untrained staff at dairy farm sites without the requirement for additional equipment.

## Data Availability Statement

The datasets for the strains used for this study can be found in the NCBI accession no. OOC91413.1, ATCC ATCC25923, ATCC19433, ATCC25922, ATCC13883, ATCC27368, ATCC27853, ATCC12386, ATCC43078, ATCC27335, ATCC19436. All other raw data supporting the conclusions of this manuscript will be made available by the authors, without undue reservation, to any qualified researcher.

## Ethics Statement

Ethical review and approval was not required for the animal study because Animal handling procedures for preparation of monoclonal antibodies were performed in compliance with institutional guidelines and followed the Act on Welfare and Management of Animals enforced in October 1973. A local ethics committee ruled that no formal ethics approval was required to conduct this research other than the above consideration. Additionally, before conducting the research, informed consent was obtained from all owners or managers of the dairy farms used in this study. Written informed consent was obtained from the owners for the participation of their animals in this study.

## Author Contributions

YN, YK, and TH substantially contributed to conception or design of the study. TH supervised all surveillance components. YN, KS, NY, KO, KF, TS, and KM contributed to acquisition, analysis, or interpretation of data. YN prepared the initial draft, figures, and tables. All authors contributed to the writing and editing of the manuscript.

### Conflict of Interest

The authors declare that this study received funding from Asahikasei. The funder had the following involvement with the study: collection and analysis. KM is an employee of Asahi Kasei Corporation and co-inventor of a patent application describing the method for detecting specific substances in milk (U.S. Patent No. 20150160212A1) but doesn't have stocks or options. The remaining authors declare that the research was conducted in the absence of any commercial or financial relationships that could be construed as a potential conflict of interest.
